# A novel nanohydroxyapatite/polyamide 66 strut for reducing subsidence after one-level anterior cervical corpectomy and fusion: a finite-element study

**DOI:** 10.1186/s40001-024-01978-2

**Published:** 2024-07-19

**Authors:** Weiyang Zhong, Ke Tang, Zhengxue Quan

**Affiliations:** 1 Department of Orthopaedic Surgery, The First Affiliated Hospital of Chongqing Medical University, Chongqing, China; 2 Orthopaedic Laboratory of Chongqing Medical University, Chongqing, China

**Keywords:** n-HA/PA66 strut, Anterior cervical corpectomy and fusion, Finite-element study

## Abstract

**Background:**

The aim of this study is to introduce a novel nanohydroxyapatite/polyamide 66(n-HA/PA66)n strut to improve biomechanical performance and reduce subsidence.

**Methods:**

One validated intact and 2 ACCF-simulated C3–C7 cervical spine models were developed (old strut: Group A, new strut: Group B). In the ACCF models, C5 underwent corpectomy and was fixed by an anterior cervical plate. Screw angles were categorized as 1 (0 ) and 2 (45 ) and divided into 4 groups, A1, A2, B1 and B2, for each model. An axial force of 74 N and a moment couple of 1.0 Nm were imposed on the C3 vertebra. The range of motion (ROM) of each segment and the stress distribution on the screw–vertebra interface, strut, and strut–endplate interface were recorded and analysed.

**Results:**

There was no significant difference in ROM between Group A and Group B during bending, extension and rotation under 74 N axial pressure. The stress concentration on the strut body in Group A was higher than that in Group B. The peak stress values at the screw–vertebral interface in Groups A1 and A2 were higher than those in Groups B1 and B2, except for during extension and lateral bending. Under axial pressure, the peak stress values at the strut body–endplate interface during bending, extension and rotation were lower in the A1 and A2 groups than in the B1 and B2 groups. The Group B model showed much higher graft stress than the Group A model.

**Conclusions:**

Based on finite-element analysis, compared with the old strut, the novel strut showed better biomechanical performance at the screw–vertebra interface.

## Introduction

Anterior cervical corpectomy and fusion (ACCF) is widely used in treating cervical diseases such as degeneration, trauma and tumours. At present, the bone graft reconstruction methods used after decompression mainly include autologous iliac or fibular bone grafts, allogeneic bone grafts, titanium mesh cages (TMCs) or nanohydroxyapatite/polyamide 66 (n-HA/PA66) strips. Although TMCs are relatively convenient to apply and are not associated with complications at the donor site, TMCs are prone to subsidence and associated with both the recurrence of neurological symptoms and potential internal fixation failure [1, 2].

n-HA/PA66 is a new nano-biomimetic composite invented and developed by our team that has achieved good results in preliminary basic and clinical studies. The fusion rates and clinical outcomes observed during the long-term follow-up for patients who underwent ACCF with a n-HA/PA66 strut were satisfactory. During the long-term follow-up of a few patients, we found that some struts had subsided and that there was a gap between the strut area and the bone on radiographs. In addition, there was no bone graft fusion around the cylinder [3]. Based on the follow-up results, the n-HA/PA66 strut needed to be improved. We further improved the design of the n-HA/PA66 strut and conducted a biomechanical comparison of the n-HA/PA66 strut and novel n-HA/PA66 strut with the anterior cervical plate in the ACCF model through a finite-element study to provide a framework for assessing changes in the mechanical properties and clinical application of the novel n-HA/PA66 strut.

## Materials and methods

This study was approved by the Institutional Review Board of The First Affiliated Hospital of Chongqing Medical University and was conducted according to the principles of the Declaration of Helsinki. All patients provided written informed consent to participate in this study before the storage of their data in the hospital database. The computed tomography (CT) images of the cervical spine were obtained from a healthy female volunteer (aged 28 years, weight 56 kg, and height 167 cm) without any cervical diseases. The CT (Siemens, Germany) scanning parameters were as follows: 120 kV, 125 Ma, layer thickness of 0.625 mm, range C1–T1. After data collection, the data were saved in standard DICOM format. The following software programs were used: Windows 7X64 Professional Edition (Microsoft, USA), Mimics 14.0 (Materialise, Belgium), Geomagic Studio 2013 (3D, USA), NX 12.0 (Siemens, Germany), and Abaqus 6.14 (Siemens, USA).

A three-dimensional model of screw plate fixation was created based on the complete three-dimensional model of the cervical spine established by NX12.0 software. The old design of the n-HA/PA66 strut (Group A) and the new design of the n-HA/PA66 strut (Group B) were compared. Group A was the old design of the n-HA/PA66 strut plus the anterior cervical screw plate fixation system. Group B was the new design of the n-HA/PA66 strut plus the anterior cervical screw plate fixation system. Screw angles were categorize as 1 (0 ) or 2 (45 ) and divided into four groups: A1, A2, B1 and B2.

**Fig. 1:**
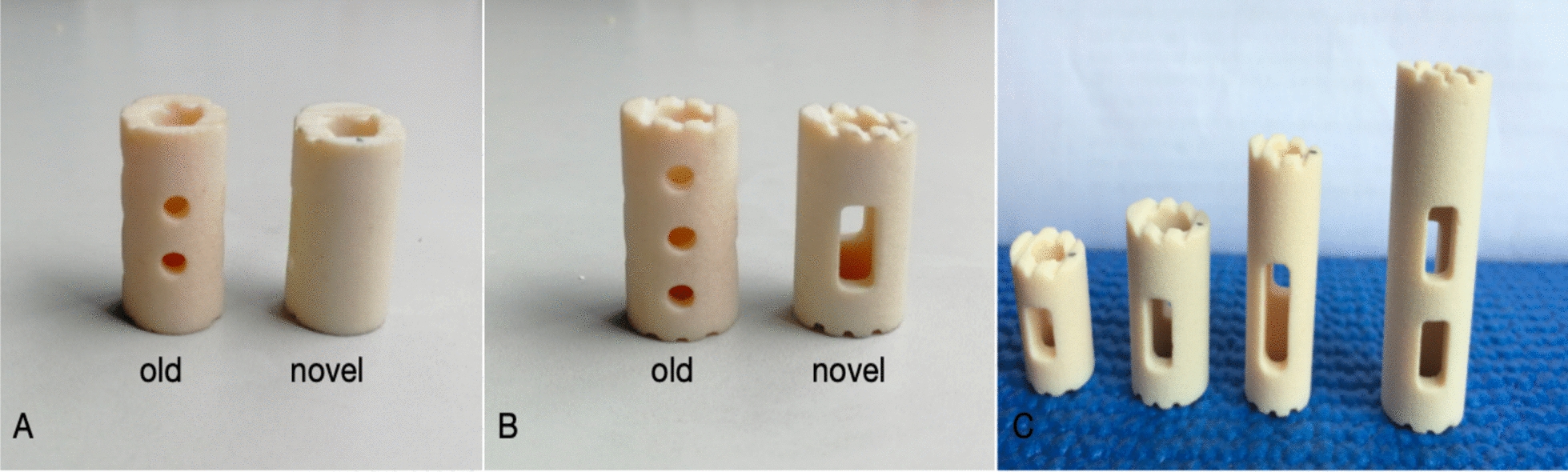
Traditional and new strut body. **A**: Front view, **B**: lateral view, **C**: various optimized models

**Fig. 2:**
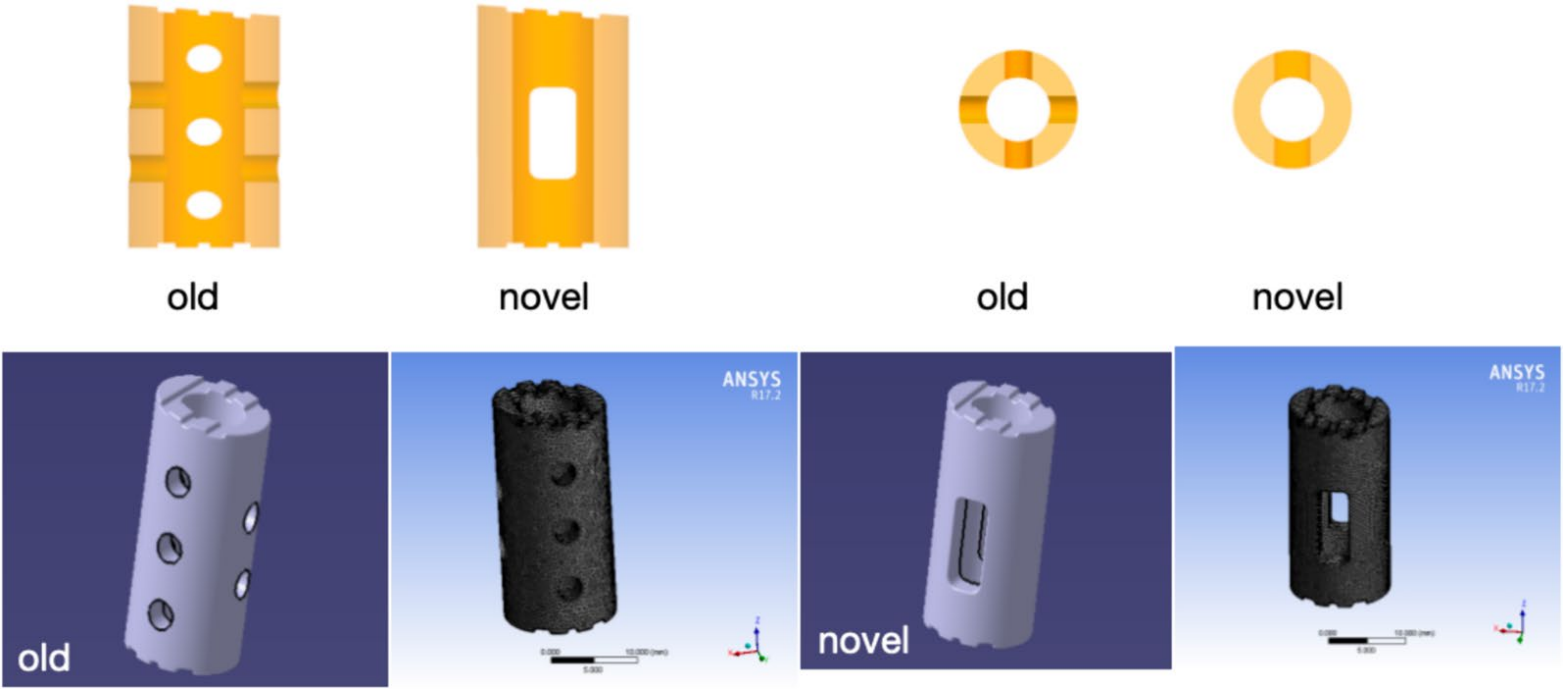
Establishment of strut models

The strut body is composed of a cylindrical body and an endplate with unique contact surfaces at both ends. The cylindrical main body is hollow (the same as before), with two small circular holes in the front and back and three small circular holes in the lateral sides. The novel design of the cylindrical body is also hollow, the front and rear four small circular holes are closed, and the left and right three small circular holes are changed to one lateral hole to further expand the contact surface between the strut body and the vertebral body to promote bone graft fusion. According to the length and width of the vertebral bodies, different models are created to show the intraoperative placement of the newly optimized strut (Figs. 1, 2).

Based on the normal cervical finite-element model, the ACCF operation was simulated by using NX 12.0 software. First, the anterior longitudinal ligament, C4–5 and C5–6 were removed, and then part of the C5 vertebral body and posterior longitudinal ligament were removed for complete decompression. The height of the decompression area was measured, and a matching geometric model of the strut was made in the NX 12.0 software. The geometric model was guided to the NX 12.0 software in assembly mode to generate a 3D finite-element model, which was added to the decompression area, and the old strut and newly designed strut were implanted between the vertebral bodies. At the same time, the fixed model of the nail board was established, and the model file was imported into Abaqus6.14 software for finite-element analysis and postprocessing. Both the upper surface of the C3 vertebral body and the upper articular surface of the model were subjected to 74 N axial pressure and 1 N·m pure couple moment to simulate the head weight and six working conditions of flexion, extension, lateral bending and rotation. Convergence analysis was performed for the maximum von Mises stress area of the fixation system, and the von Mises stress distribution was recorded and analysed at the screw–vertebral interface, strut interface, and strut–endplate interface. The maximum stress value, stress nephogram and interbody ROM were obtained, and comparative analysis was conducted among the groups.

## Results

**Fig. 3:**
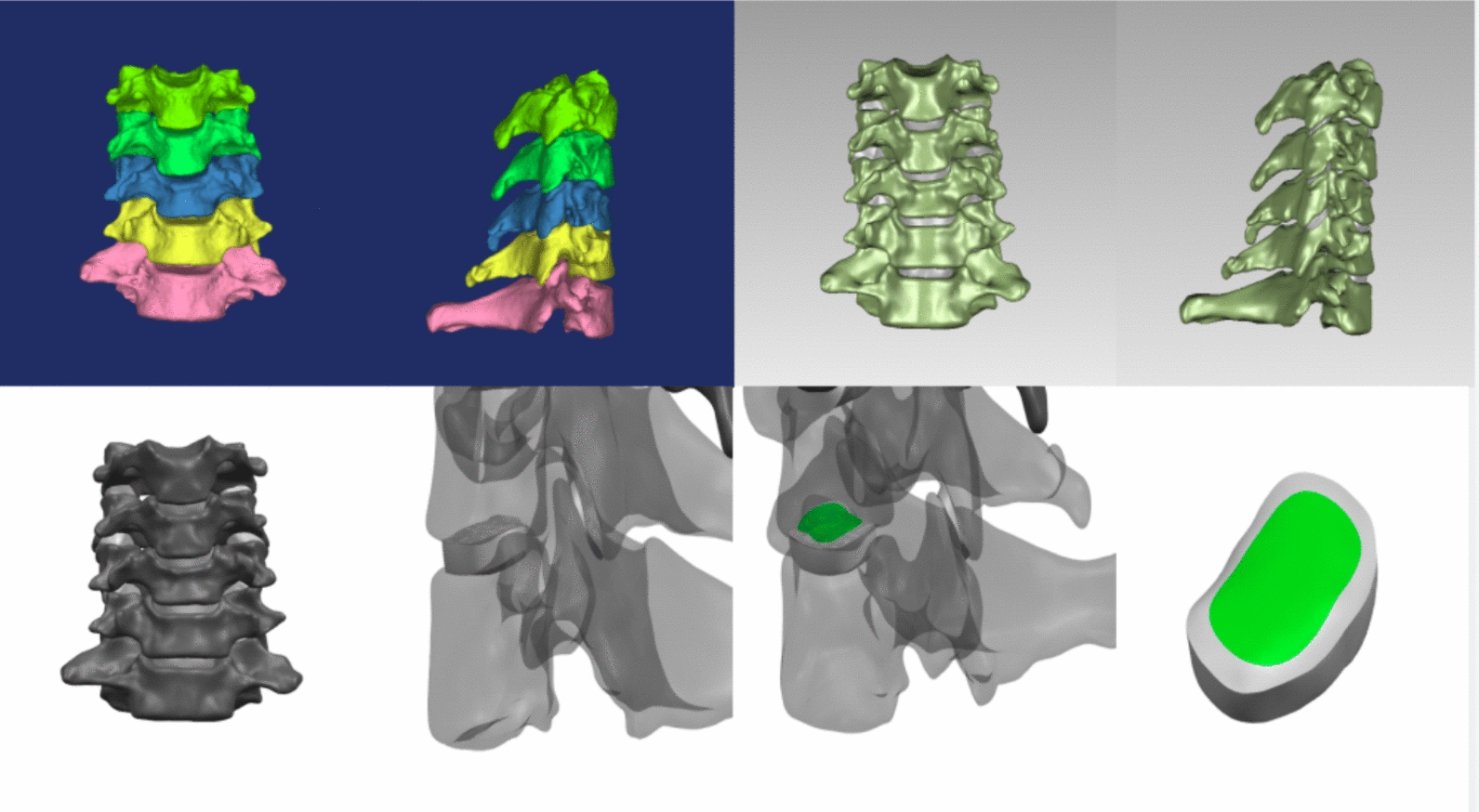
Establishment of a three-dimensional model of the lower cervical spine

**Fig. 4:**
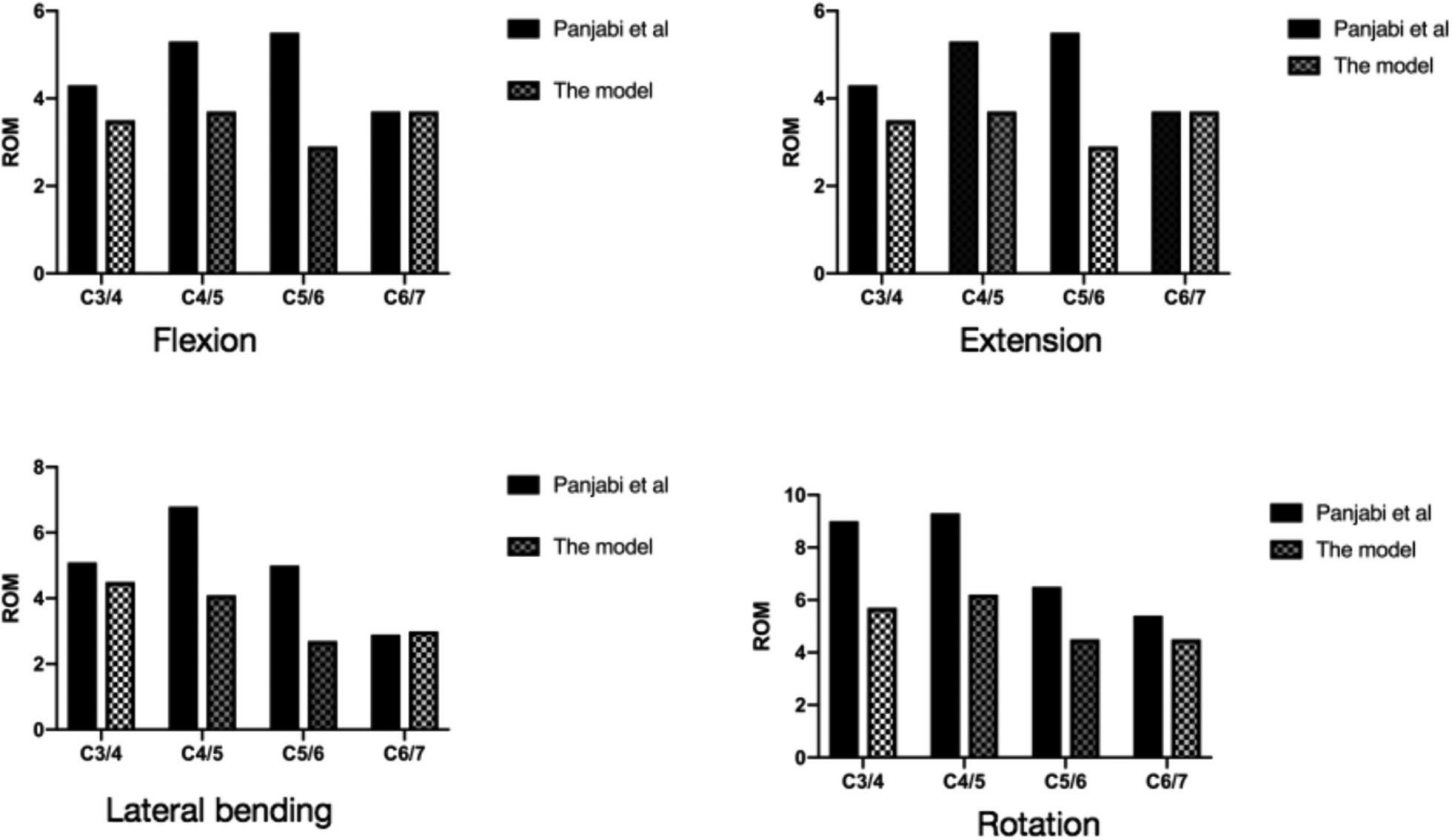
Verification of the ROM of the FE model of the lower cervical spine

A normal C3–7 nonlinear finite-element model was successfully established, which simulated the three-dimensional structures of cortical bone, cancellous bone, annulus fibrosus matrix and fibres, nucleus pulposus, endplate cartilage, facet cartilage and related ligaments, with a total of 296,398 units and 400,670 nodes (Fig. 3). The interbody ROM of the complete group of models was compared with the experimental results reported by Panjabi et al. (Fig. 4). Under six working conditions of flexion, extension, lateral bending and rotation, the consistency was good, which verified the effectiveness of this model.

**Fig. 5:**
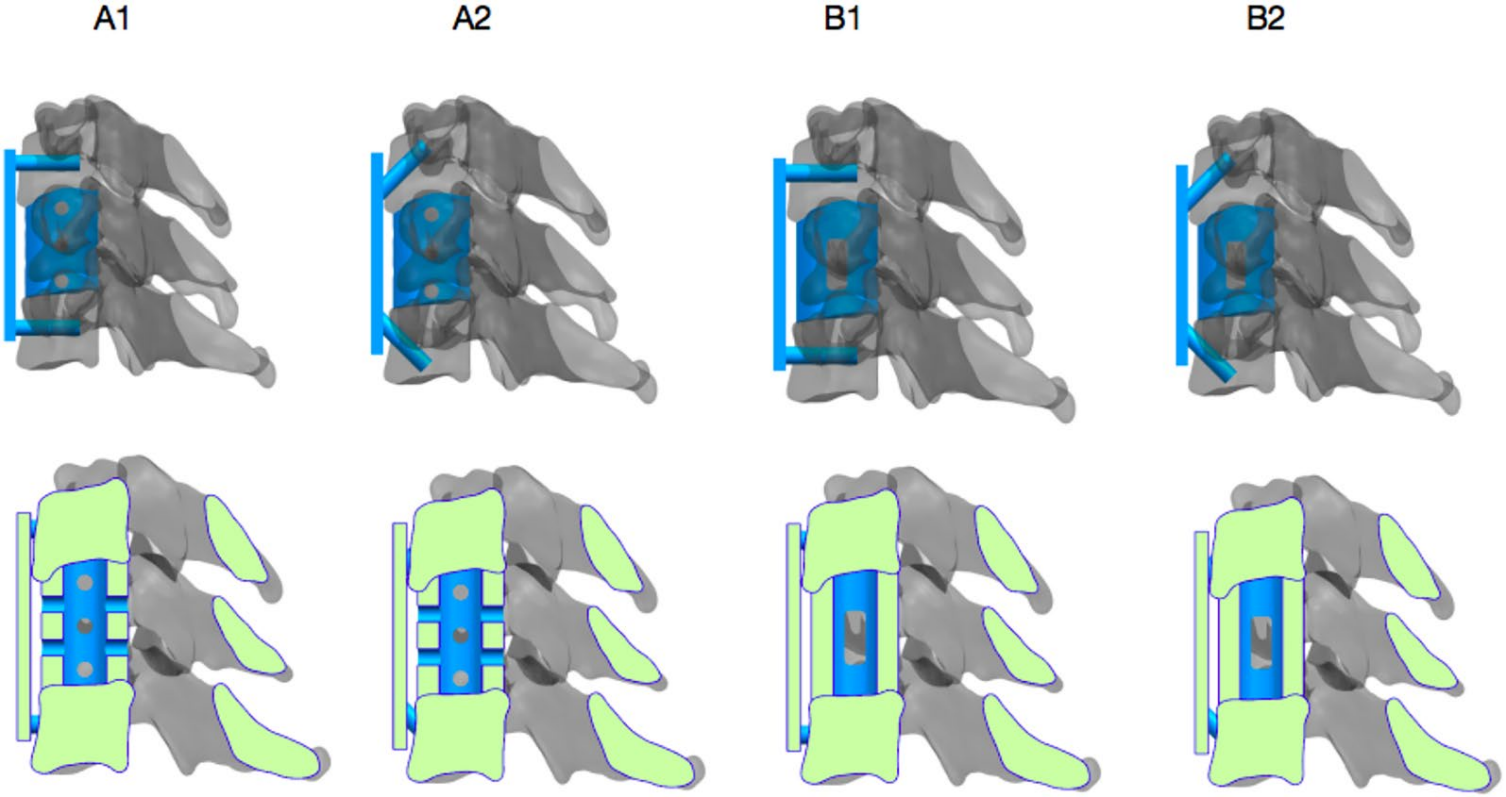
Stress nephogram of extension–flexion conditions and under 74 N axial force

**Fig. 6:**
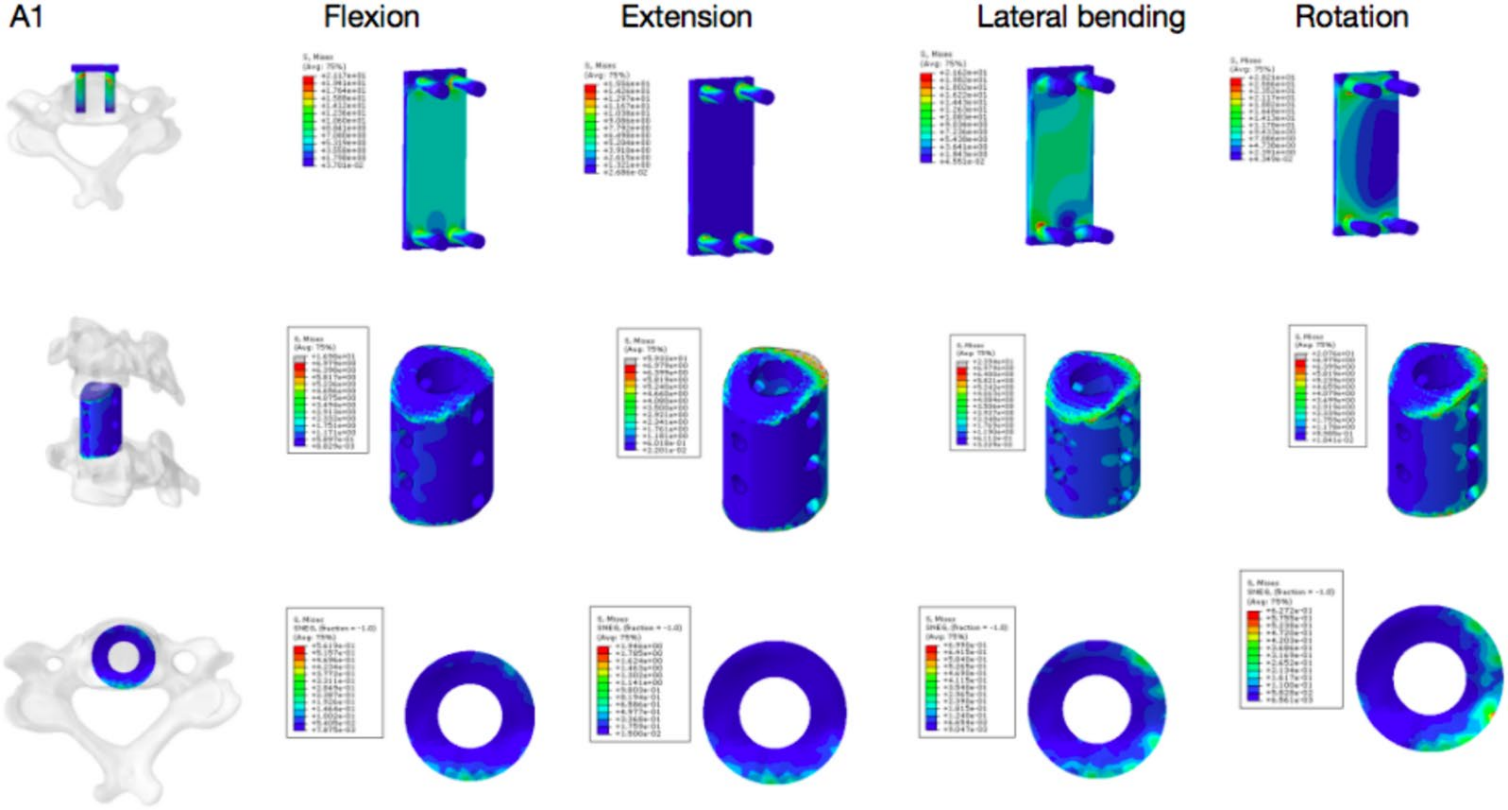
A1 group under 74 N axial force

**Fig. 7:**
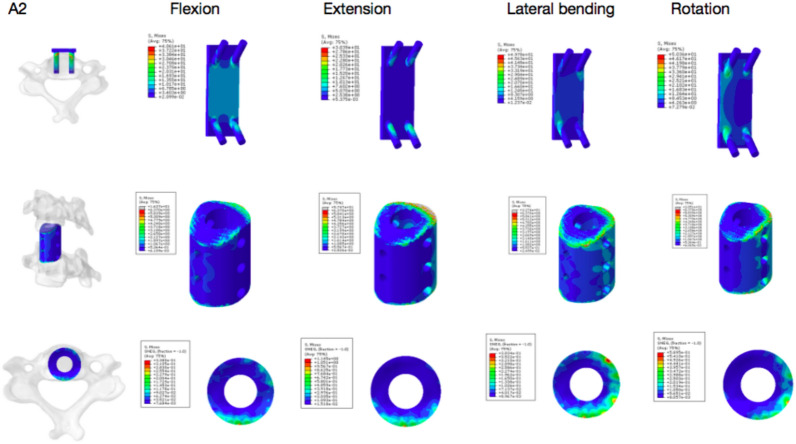
A2 group under a 74 N axial force

**Fig. 8:**
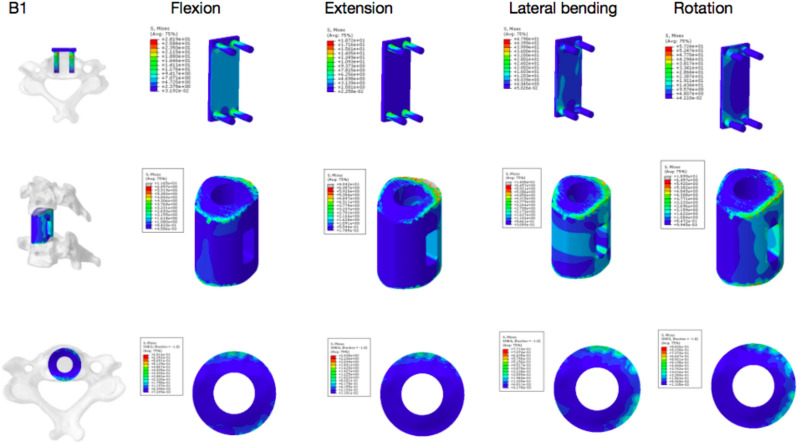
B1 group under the condition of a 74 N axial force

**Fig. 9:**
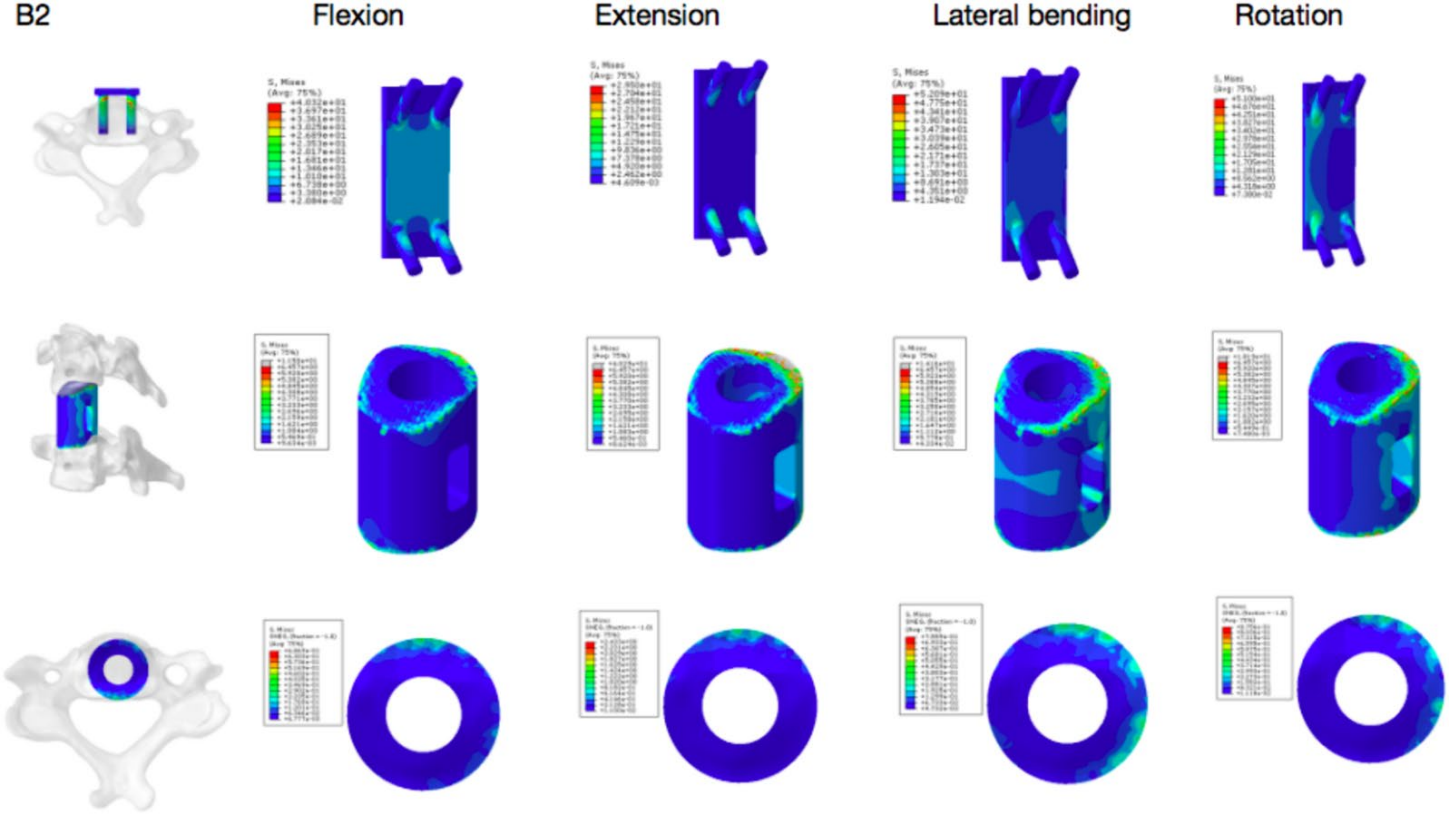
Group B2 under 74 N axial force

The results of finite-element analysis showed that there was no significant difference in ROM between Group A and Group B under 74 N axial pressure during bending, extension and rotation. The stress concentration of Group A was higher than that of Group B. The peak value at the screw–vertebral interface of Groups A1 and A2 was higher than that of Groups B1 and B2 except for during extension and lateral motion. Under axial pressure, the peak stress at the strut–endplate interface during bending, extension and rotation was lower in the A1 and A2 groups than in the B1 and B2 groups. Group B was subjected to higher graft stress than Group A (Figs. 5, 6, 7, 8, and 9).

## Discussion

ACCF is widely used in treating cervical diseases such as degeneration, trauma and tumours because of its advantages, such as a large surgical field, easy operation, thorough decompression and accurate curative effect. TMCs are commonly used in reconstruction, and the long-term follow-up results show that there are still some defects, such as subsidence leading to recurrent neck pain, neurological impairment, or even failure of internal fixation [1, 2]. The n-HA/PA66 invented and developed by our team is a new nano-biomimetic material. After frequent clinical applications, the initial and long-term follow-up showed that the fusion rate and clinical results of the n-HA/PA66 strut were satisfactory. However, we found that part of the strut subsided, a gap appeared in the cylinder area on radiograph, and there was no bone graft fusion or bone graft fusion around the cylinder [1–3]. Based on the follow-up results, the design of the n-HA/PA66 strut still needs to be improved to achieve better bone fusion. We further improved the design of the n-HA/PA66 strut. Therefore, based on old biological materials, this study designed a new face of a strut. The columnar body of the new strut body is hollow, similar to the old strut body, and the lateral small round holes are replaced with a rectangular lateral hole to further expand the contact surface between the strut body and the vertebral body to achieve better biomechanical performance and to better distribute stress, avoiding postoperative subsidence and increasing the biomechanical stability at the surgical level [4–7].

In this study, we successfully established a C3–7 complete state nonlinear finite-element model, which accurately simulated the cortical bone, cancellous bone, facet cartilage, endplate cartilage, nucleus pulposus, intervertebral disc annulus fibrosus matrix, annulus fibrosus fibres and intervertebral ligaments of various parts. The intervertebral ligament was assigned nonlinear stiffness to better simulate the nonlinear motion characteristics of the cervical vertebra to be closer to the real cervical vertebra. After the model was established, it was verified with the experimental model in the study by Panjabi et al. [4–11], and the results showed a good anastomosis, indicating that the finite-element model has good effectiveness and reliability, can objectively reflect the biomechanical properties of the lower cervical vertebra, and can be used for further study. The results showed that there was no significant difference in ROM between Group A and Group B under 74 N axial pressure during bending, extension and rotation. The stress concentration of the artificial vertebral body in Group A was higher than that in Group B. The peak stress values at the screw–vertebral interface of Groups A1 and A2 were higher than those of Groups B1 and B2 except for during extension and lateral bending, indicating that Group B had stronger resistance to bending and rotational pressure and a stronger ability to pull out and rotate screws than Group A. According to Wolff's law, the formation of new bone depends on the strain interval of bone tissue. A certain pressure will promote the formation of bone, while no pressure will promote the absorption of bone. In this study, the peak values of axial pressure and stress at the strut–endplate interface during bending, extension and rotation were lower in the A1 and A2 groups than in B1, and the Group B model of B2 showed much higher bone graft stress than the Group A model. Although the optimal stress needed for strut–endplate fusion is not yet known, Group B may provide a new way to improve ACCF fusion rates. At the same time, the optimized lateral space of the strut body is also fused with the vertebral body to improve the fusion rate between the strut and the vertebral body bone graft to improve the biomechanical stability of the cervical spine [12–18].

The finite-element model study results showed that the Group B model has better stability than the Group A model in terms of structure composition, its risk of internal fixation fracture is lower, and there is no significant difference between Group A and Group B in terms of the influence on the mobility of adjacent segments. At the same time, this model can simulate the geometric structure of normal cervical vertebra and ACCF surgery and obtain data that can be used to analyse the reliability and application effect of the internal fixation device and provide technical support for the improvement of the design of the internal fixation device.

Although the finite-element study is an effective method for assessing cervical spine biomechanics, it still has some limitations. The results were only representative of conditions imposed upon the model, and the human body itself is organized with biological activity. There is an aging process, and the finite-element model can only respond to the mechanical properties of the body at a specific point in the aging process. At some point, the whole process cannot be reflected. These deficiencies may affect the accuracy of the experimental results to some extent.

## Conclusions

Based on finite-element analysis, compared with the old strut, the novel strut showed better biomechanical performance at the screw–vertebra interface and strut. In the future, further biomechanical studies, such as comparative studies of the n-HA/PA66 strut with conventional struts and animal clinical studies, need to be performed.

## Data Availability

The data sets generated and/or analysed during the current study are not publicly available due to the importance of upholding ethical standards in terms of protecting patient data and ensuring anonymity but are available from the corresponding author upon reasonable request.
